# Integration of clinical and basic sciences in concept maps: a mixed-method study on teacher learning

**DOI:** 10.1186/s12909-015-0299-0

**Published:** 2015-02-18

**Authors:** Sylvia C Vink, Jan Van Tartwijk, Jan Bolk, Nico Verloop

**Affiliations:** 1 ICLON, Graduate School of Teaching & Leiden University Medical Centre, Wassenaarseweg 62A, 2333 AL Leiden, The Netherlands; 2 Centre for Teaching and Learning, Educational Development and Training, Faculty of Social and Behavioural Sciences, Utrecht University, PO Box 80.140, 3508 TC Utrecht, The Netherlands; 3 Leiden University Medical Centre, PO Box 9600, 2300 RC Leiden, The Netherlands

**Keywords:** Curriculum development, Instructional design, Educational intervention, Clinical knowledge, Analysis of content, Concept mapping, Cooperative learning, Teacher learning, Design-based research, Clinical reasoning

## Abstract

**Background:**

The explication of relations between clinical and basic sciences can help vertical integration in medical curricula. Concept mapping might be a useful technique for this explication. Little is known about teachers’ ability regarding the articulation of integration. We examined therefore which factors affect the learning of groups of clinicians and basic scientists on different expertise levels who learn to articulate the integration of clinical and basic sciences in concept maps.

**Methods:**

After a pilot for fine-tuning group size and instructions, seven groups of expert clinicians and basic scientists and seven groups of residents with a similar disciplinary composition constructed concept maps about a clinical problem that fit their specializations. Draft and final concepts maps were compared on elaborateness and articulated integration by means of t-tests. Participants completed a questionnaire on motivation and their evaluation of the instructions. ANOVA’s were run to compare experts’ and residents’ views. Data from video tapes and notes were qualitatively analyzed. Finally, the three data sources were interpreted in coherence by using Pearson’s correlations and qualitative interpretation.

**Results:**

Residents outshone experts as regards learning to articulate integration as comparison of the draft and final versions showed. Experts were more motivated and positive about the concept mapping procedure and instructions, but this did not correlate with the extent of integration fond in the concept maps. The groups differed as to communication: residents interacted from the start (asking each other for clarification), whereas overall experts only started interaction when they had to make joint decisions.

**Conclusions:**

Our results suggest that articulation of integration can be learned, but this learning is not related to participants’ motivation or their views on the instructions. Decision making and interaction, however, do relate to the articulation of integration and this suggests that teacher learning programs for designing integrated educational programmes should incorporate co-construction tasks. Expertise level turned out to be decisive for both the level of articulation of integration, the ability to improve the articulated integration and the cooperation pattern.

## Background

Medical curricula are intended to help students to relate clinical and basic science knowledge. Localizing underlying basic science mechanisms allows teacher and students to focus on relevant relations with clinical phenomena [1]. To develop a curriculum that addresses this so-called vertical integration requires the articulation of basic science mechanisms and their relations with clinical concepts, because designing an educational programme requires a clear view on what knowledge should be incorporated [2]. Teachers’ tacit knowledge is of little help to develop an instructive curriculum [1,2], and this holds true for both clinical or basic science tacit knowledge. However, clinical teachers, often experienced clinicians, are used to rely on illness scripts when analysing patient cases [3], using so-called chains of practice [1]. Their basic science knowledge pertains to the underlying mechanisms of understanding these patient cases, but tends to remain inactivated when they analyse patient cases that to them are not complicated [4,5]. For the development of an integrated curriculum, teachers should be able to decide which clinical and basic science concepts, and which relations between them, should be incorporated in the programme in order to design assignments, choose relevant patient cases and guide student discussions. Therefore, the study presented here investigated how medical teachers can be instructed to make their integrated clinical and basic science knowledge explicit. Concept mapping is a technique by which to explicate and share knowledge [6,7]. The resulting concept maps contain networks of hierarchically ordered and linked concepts. Concept mapping is recommended as a means to elicit tacit knowledge [8] and thus might help medical teachers to articulate relations between clinical and basic science knowledge [1,9].

### Teachers’ learning to articulate integration

The articulation of integration of clinical and basic sciences is not receiving much attention in teacher learning programmes in the medical domain [10]. When it comes to teachers’ ability to explicate the integration of clinical and basic sciences, looking at three prevalent views on learning [11] might help to understand how teachers improve the articulation of integration (cf [12]): a cognitive view, emphasizing teachers’ knowledge as a source for improving teaching practice; a constructivist view, stressing teachers’ learning process as an active interpretation process of new information based on teachers’ own knowledge and experiences; and a third view emphasizing the cooperative aspects of teacher learning, that is, teachers learning with and from other teachers. For concept mapping as a means for teacher learning, these three views pertain to different aspects. The cognitive view addresses the concept maps themselves, which reflect the integration of clinical and basic science concepts that teachers are able to explicate, and hence designate the integration they are likely to apply in their teaching practice. Here, the instructions guiding the teachers in constructing the concept map should be taken into account, because they influence what teachers articulate in the concept map [13]. From a constructivist point of view, it is not the concept maps, but the process of concept mapping that is vital [7]. Examining draft and final versions of concept maps and teachers’ views on the activity of concept mapping helps us to understand how medical teachers apply the concept mapping instructions and how they improve the articulation of integration of clinical and basic sciences [7,14,15]. The third view on teacher learning also focuses on the process of learning, but highlights the importance of cooperation. Although some studies recommend involving more than one constructor in the construction of concept maps for educational purposes, in none of these studies are concept maps constructed *jointly* [14,16-18]. For the development of integrated curricula, communication between clinicians and basic scientists is deemed decisive [19], in particular because they have different views on which basic science concepts should be incorporated in a medical education programme [20]. Thus, cooperative learning, with its strong emphasis on communication, could be helpful for the articulation of integration. Due to the information gaps in mixed groups [21], establishing the relations between clinical and basic sciences is expected to be easier than when teachers construct the maps individually. Research on the cooperation between teachers can illuminate how teachers learn [22], and so could contribute to our understanding of how medical teachers can explicate the integration of clinical and basic sciences in concept maps and of the factors that affect their ability for articulation.

### The process of teacher learning

Concept mapping with the aim to visualize integration of clinical and basic sciences is still in its infancy. Although the technique is recommended for this purpose [1,9,17], there are not so many examples of concept maps that show the relations between clinical and basic sciences. Evidently, the general instructions for concept mapping as proposed by Novak [7] do not automatically lead to concept maps that visualize vertical integration. Therefore, specific concept mapping instructions that help medical teachers to articulate the integration of clinical and basic sciences seem required. The impact of such concept mapping instructions might depend on expertise level of the constructors. In a previous study (Vink SC, Van Tartwijk J, Bolk JH, Verloop N, Gosselink MJ: Consistent variations between concept maps constructed by expert groups and residents, submitted) we found that residents were able to articulate the integration of clinical and basic sciences in concept maps to a significantly greater extent than experts. A cognitive explanation for this could be that due to their clinical experience, experts’ basic science knowledge becomes encapsulated by clinical higher order concepts, whereas the basic science knowledge of residents plays a more overt role in the understanding of clinical problems, cf. [4,5]. Although experts can still relate these encapsulated basic science concepts to clinical concepts when they are presented [23] to them, detailed relations between clinical and basic science concepts seem to be irrelevant for understanding a clinical problem in educational settings. It was also suggested that group dynamics and their reflection in the communication could account for the level to which expert groups and resident groups articulate their knowledge. If this is the case, the expertise level of the constructor groups could then account for differences in the concept mapping processes, i.c. the dynamics of cooperation [22] and consequently for differences between the concept maps. We therefore scrutinized the process of concept mapping and searched for the factors that account for the articulated integration in the concept maps and affect thus the learning process of interdisciplinary groups of clinicians and basic scientists at different expertise levels. Insights into the factors that facilitate or hinder the articulation of integration can be used to refine the concept mapping instructions, in order to instruct teachers effectively. So far, research has focused on the concept maps themselves, i.e., taking a cognitive point of view [14,24]. Our focus in this study was on the process of concept mapping, thereby exploring constructivist and cooperative learning approaches.

## Methods

### Participants and procedure

Seventeen groups, all composed of both clinicians and basic scientists working at the Leiden University Medical Centre, participated in the experiments, including the pilot experiments. With the invitation for participation, aims of the sessions, procedure and time investment were explained. Acceptance of the invitation was interpreted as participants’ consent. Ten groups were designated as ‘experts’: the participants had at least five years’ experience as clinician or basic scientist and were involved in preclinical and/or clinical education. Two groups consisted of five, one group of two, and seven groups of three experts. To examine the influence of expertise level, seven resident groups with a disciplinary composition equivalent to that of the seven groups of three experts were included (see Tables 1 and 2 for an overview of the participants). Each group constructed a concept map about a clinical problem that fitted the disciplinary composition of the group, e.g., a surgeon, a pathologist and a general practitioner constructed a concept map about blood in faeces, and a lung specialist, a specialist in infectious diseases and an immunologist constructed a map about coughing. In order to minimize the influence of content bias on the findings, concept maps of eight different clinical problems were constructed.

**Table 1:** **Composition of groups of the pilot**

Concept map	Discipline of each participant
Cough	GP
	oto-rhino-laryngology (focus anatomy)
Hypertensia	physiology cardiac diseases
	physiology
	internal diseases
	internal diseases
	nephrology
Proteinuria	nephrology
	nephrology
	pathology
	physiology
	internal diseases

**Table 2:** **Composition of both expert and resident groups**

Blood in faeces	GP
	pathology
	surgery
Chronic abdominal pain	radiology/anatomy
	gynaecology
	internal diseases
Cough	infectious diseases
	immunology
	lung diseases
Diarrhoea	anatomy
	gastro-internal diseases
	infectious diseases
Diarrhoea	gastro-internal diseases
	microbiology
	surgery
Painful joints	immunology
	rheumatology
	surgery
Proteinuria	gynaecology
	Pathology
	Nephrology

All groups consisted of 3 participants.

The aim of the concept mapping sessions was to construct a concept map which included all relevant concepts and relations needed for clerks to understand the clinical problem at hand. Because the focus of the study was the articulation of integration, the groups were asked to explicate all information relevant for clerks, without bothering about the actual use of the concept map in medical education, either for curriculum planning or as a help for students. The concept maps were constructed in at least two sessions, as recommended by Novak [25]. We considered the draft made during the first session to be an intermediate state in the ability to articulate integration, which was further developed during the second session [1]. In the first session, the groups were guided step by step through concept mapping instructions that included directives intended to encourage them to articulate the integration of clinical and basic sciences. First they were instructed to contribute and discuss concepts that were particularly relevant from the perspective of participants’ own disciplines. To keep an overview of these concepts, the groups could organize them in any way they wanted. The second instruction focussed on organization: the groups explored both clinical concepts and basic science concepts as higher order concepts by which to organize basic science and clinical concepts, respectively. Subsequently, the groups were encouraged to explore any other relations between clinical and basic science concepts and to link them. As a final step, the participants had to explain two complex patient cases in order to check whether the concept map was comprehensive enough. The draft versions were constructed with the aid of post-it notes and large sheets of paper. We expected this “physical” way of constructing to enhance communication, particularly in groups in which participants met each other for the first time. This would thus contribute to the learning process [21]. After the first session, the first author digitized the draft concept maps by means of *Inspiration*@, a software tool for concept mapping. Approximately a month later, in a second group session, participants were asked to check whether any mistakes had been made during digitization, and to review and refine the ordering and relations between the concepts in order to improve the articulation of the knowledge relevant for clerks. A researcher was present to explain this aim and to remind the groups to use the hand-out with the instructions but they were not guided through the instructions, as they were in the first session. Scheduling of the second session depended on the diaries of the group members. The Institutional Board of Leiden University Medical Centre, where the concept maps were constructed, provided ethical approval for the study.

Before the actual experiment started, we conducted pilots with groups of five and two participants to find the optimal group size and instructions. In cooperative learning, group size has been associated with different interaction patterns [26]. We assumed that larger groups, with consequently more disciplines, would mean a greater challenge to bridge the gaps between the disciplines [27], whereas small groups implied a less challenging task to articulate integration. This might be reflected in the communication patterns [22,27]. When group size and instructions had been optimized, leading to a construction process that the participants experienced as feasible, we were able to investigate the context variable ‘expertise level’ [28]. The optimal group size turned out to be three.

### Data collection and analysis

Three data sources were used: the draft and final versions of the concept maps (to track improvement of articulation during the concept mapping process), a questionnaire (to examine the perceived usefulness of the instructions) and video tapes of the sessions combined with field notes (to analyse cooperation). After each first session of the pilot groups and the expert groups, we discussed the instructions with each group to check feasibility and clarity, so that we could adapt them to practical needs. After the pilot, there were no major adaptations of the instructions.

#### Concept maps

**Figure 1:**
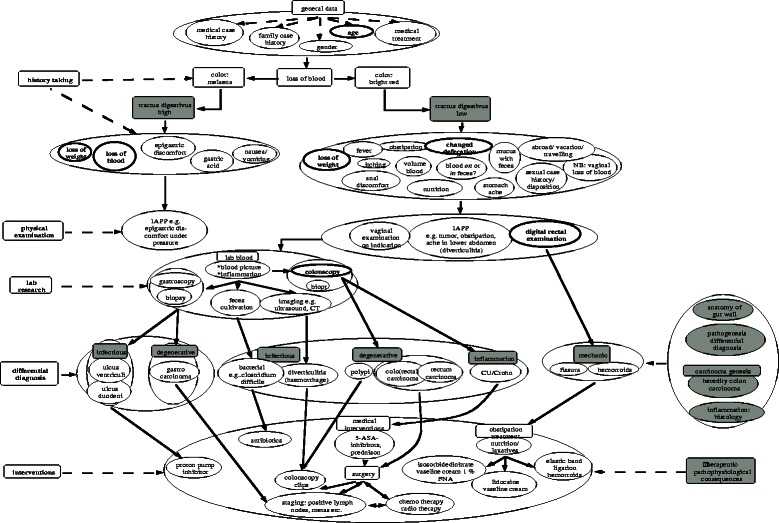
**Resident concept map about blood in faeces, constructed by a GP, a surgeon and a pathologist.** Clinical concepts are white, basic science concepts are grey coloured. Features of integration: links (e.g. ‘digital rectal examination’ linked with ‘mechanic’) and basic science concepts subsuming several clinical concepts (e.g. ‘tractus digestivus high’ subsuming five clinical concepts). Rectangular shapes indicate umbrella concepts. Oval shapes indicate any other concepts.

We measured both the elaborateness of the draft and final versions of the concept maps in terms of number of clinical and basic science concepts, and features that measured the articulated integration of clinical and basic sciences (see Figure 1 for examples). These features had been developed in a previous study to describe differences in articulated integration. The interrater reliability in that study turned out to be sufficient (a mean Cohen’s kappa of .95 (Vink SC, Van Tartwijk J, Bolk JH, Verloop N, Gosselink MJ: Consistent variations between concept maps constructed by expert groups and residents, submitted) to justify having one researcher coding the draft versions in the present study. The concept maps of the pilot were left aside, because the instructions were modified after the pilot sessions. The differences in articulated integration between the first and the second session were measured by performing a *t*-test for two related samples on the analysis of the draft and final versions.

#### Questionnaire

In order to incorporate participants’ points of view on what factors facilitated the articulation of clinical and basic science knowledge in concept maps, after the first session we asked them to fill in a questionnaire focusing on the usefulness of the instructions. The items were answered on a five-point Likert scale ranging from ‘I do not agree’ to ‘Agree’ or ‘Very low’ to ‘Very high’ in case of the questions on motivation. In the version filled in by the residents an additional four items were used to question the different ways of ordering the concepts map in detail, because the residents were discussing the different ways of ordering to a much greater extent than the experts did. In order to examine the impact of the sessions on motivation, *t*-tests were run on the questions concerning motivation. Because the integration as articulated in the expert concept maps differed significantly from that in the resident concept maps, ANOVAs were used to investigate whether these groups also differed regarding the perceived usefulness of the instructions. Additional Pearson’s correlations were performed to examine whether the means of participants’ motivation, their views on procedure and instructions and their satisfaction with the concept map were related to the articulation of integration measured by the number of links.

#### Video tapes and field notes

We gathered data by means of video tapes and field notes of the pilot sessions, and the first sessions of eleven three-participant groups to examine cooperation within the groups. A first rough qualitative analysis was conducted by means of a checklist matrix structured along the concept mapping instructions [29]. Per instruction and per participant, notes were made of questions, answers to questions, subjects of discussion, positive and negative remarks and motivations that participants gave for their contributions, in order to map out the communication in the group. Moreover, per instruction we wrote observations that pertained to the whole group, such as how much effort it took to apply the instruction and make decisions, and added quotes to illuminate the notes. One tape was analysed by two researchers in order to cross-check the interpretation and to fine-tune the checklist matrix. We felt one researcher was sufficient for video analysis, because this was triangulated by data from the questionnaires and the draft and final concept maps. A summary of the notes per instruction was briefly discussed with the groups of participants during the second session, to check whether they recognized the findings. We grounded categories in the data in different rounds of analysis, thus clustering the categories that could describe the video tapes in a qualitative way. Eventually, the data were clustered into 1. motivation, 2. exchange of information, 3. interaction and 4. the decision-making process. These categories combined some of the communication patterns as described by Weinberger & Fischer [22] and some conditions for effective cooperative learning [21], which we interpreted as a validation of the categories for analysis. In Table 3 the categories are presented in detail.

**Table 3:** **Coding categories used for the analysis of the video tapes and the field notes**

Concept map	Discipline of each participant	
Category	Description	Examples
Motivation	Positive and negative drive to adopt concept mapping.	It is great fun, this way of working. (E)
	Understanding of the goal of the cooperative learning task in order to stay on track.	My enthusiasm is reduced because I still do not understand the goal of concept mapping. (R)
Exchange of information	Explanations and explications without involvement from others, e.g., explications of the participant’s own contribution to the concept map	The basic science categorization is good to know but you should not really apply it. (E)
		For me, the concept map is upside down. (E)
Interaction	Active involvement reflected in questions participants ask each other, asking for and giving clarifications	I do not know whether this results in blood in faeces. You know that. (R)
		Now I am completely confused: how do you use secretor and osmotic? Up to 2 hours ago, it was our main device. This distinction can’t be that weird? (R)
Decision making	Negotiations about how to structure the concept map, implying what to adopt in the map.	Let’s distinguish pathogenesis and pathophysiology. Okay, this categorization does not commit us to anything. (E)
		Let’s stop with expanding the concept map. Every concept covers more detailed concepts. (E)

E = Expert.

R = Resident.

## Results

### Learning expressed by draft and final concept maps

Table 4 presents the differences between the draft and final versions of the concept maps. In the second session, participants added more (especially clinical) concepts to the concept map, and articulated integration to a significantly higher extent via links between clinical and basic science concepts, and via basic science concepts subsuming clinical concepts. Additional analysis comparing resident and expert concept maps revealed that only the residents were responsible for the significant improvements in articulated integration.

**Table 4:** **Differences between draft and final versions of the concept maps**

	Draft		Final		
	N = 14***		N = 14***		
	Mean	SD	Mean	SD	t
*Clinical concepts*	*67.1*	*15.2*	*78.1*	*19.9*	3.45**
Experts	60.4	12.9	70.7	19.7	1.60
Residents	73.6	55.2	85.6	18.3	7.19**
*Basic science concepts*	*18.6*	*8.5*	*23.7*	*16.1*	*1.34*
Experts	18.4	8.3	19.1	7.6	0.36
Residents	18.6	9.4	28.3	21.3	1.31
*Clinical concepts subsuming basic science concepts*	*0.6*	*1.2*	*1.8*	*3.0*	*1.42*
Experts	0.0	0.0	2.8	3.9	1.94
Residents	1.1	1.6	0.7	1.5	-1.44
*Basic science concepts subsuming clinical concepts*	*4.3*	*4.8*	*6.0*	*5.3*	3.07**
Experts	1.1	2.3	2.6	4.4	1.64
Residents	7.4	4.6	9.4	3.9	2.65**
*Links between clinical and basic science concepts*	*12.4*	*9.0*	*15.4*	*10.3*	3.05*
Experts	5.1	3.4	6.9	4.8	1.77
Residents	19.7	6.2	24.0	6.0	2.61*

*p < 0.05.

**p < 0.01.

***7 expert concept maps and 7 resident concept maps.

### Participants’ views on concept mapping

Regarding participants’ views on procedure and instructions we distinguished between experts and residents, for their concept maps differed significantly in the articulation of integration. The reliability of the 20 items used in the analysis was satisfactory (Cronbach’s alpha .87) or high (Cronbach’s alpha .93) for the residents’ version with 24 items. Overall, experts were significantly more positive about the procedure and instructions of concept mapping than residents; seven out of twenty questions showed a significant difference between the two groups, as Table 5 shows. The experts really enjoyed the concept mapping sessions; their motivation increased significantly (p < .05), whereas residents’ motivation grew no more than slightly. The residents were significantly more positive about the instruction to order the concepts along clinical concepts than about the instruction to order the concepts along basic science concepts (p < .05) (t-values not shown in Table 5). Participants’ motivation, their view on procedure and instructions and their satisfaction with the concept map were significantly related with the extent of articulated integration in the concept maps measured by links. However, this correlation was negative (see Table 6).

**Table 5:** **Differences between experts and residents concerning their view on concept mapping procedure and instructions**

	Residents		Experts		*F*
	N = 19		N = 21		
	Mean	SD	Mean	SD	
Before this session. my motivation to participate was	3.4	0.5	3.8	0.8	3.219
After this session. my motivation to participate was	3.5	0.8	4.2	0.6	13.139**
Procedure: making a concept map					
Is feasible	3.4	0.8	3.8	0.8	2.172
Is inspiring	3.9	0.7	4.2	0.5	3.853
Is a good way to assemble concepts of various disciplines	3.9	0.6	4.4	0.6	5.127*
Enhanced my understanding of what knowledge should be incorporated in the educational programme	3.6	0.8	3.7	0.8	0.303
Facilitates multidisciplinary cooperation	3.6	0.7	4.0	0.7	2.828
I enjoyed the multidisciplinary way of working	4.0	0.7	4.6	0.7	7.241*
	3.3	0.8	3.6	0.9	1.086
Consists of logical steps					
Time investment and result are balanced	3.2	1.0	3.5	1.0	3.296
	**3.6**	**0.4**	**4.0**	**0.4**	7.302**
**Total**					
Instructions					
The introduction was understandable	3.9	0.8	4.2	O.7	1.984
	4.0	0.7	4.3	0.7	2.396
Collecting concepts & first categorization was useful					
Collecting concepts & first categorization was understandable	3.8	0.9	4.1	0.8	0.629
Ordering was useful	3.7	0.7	4.5	0.6	15.366**
Ordering was understandable	3.7	0.9	4.3	0.7	5.372*
Linking concepts was useful	3.4	1.1	4.0	0.7	3.008
Linking concepts was understandable	3.8	0.7	4.0	0.8	0.445
Using patient cases was useful	3.8	0.6	4.4	0.8	5.338*
Using patient cases was understandable	3.9	0.8	4.3	0.7	2.069
**Total**	**3.8**	**0.6**	**4.2**	**0.4**	7.443**
Overall					
I am satisfied with this concept map	3.9	0.8	4.4	0.7	5.065*
Ordering:					
Along basic science concepts is useful	3.3	1.0			
Along basic science concepts is understandable	3.6	1.1			
Along clinical science concepts is useful	4.1	0.6			
Along clinical science concepts is understandable	4.0	0.8			
	**3.7**	**0.7**			
**Total**					

*p < 0.05.

**p < 0.01.

**Table 6:** **Pearson’s correlations between participants’ motivation**

	Motivation	Mean score on procedure	Mean score on instructions	Satisfaction with the concept map
Number of links	-.506**	-.548**	-.624**	-.468

**p < 0.01.

Views on concept mapping procedure and instructions and satisfaction with the concept map with integration measured by number of links.

### Cooperative learning

After clustering the data from the video tapes and the field notes, four categories emerged: 1. motivation, 2. exchange of information within the group, 3. interaction between participants and 4. the decision-making process. Quotations below have been translated from Dutch to English, and expertise level is indicated by (E) for experts and (R) for residents in order to illustrate the impact of the context variable ‘expertise’.

#### Motivation

Regarding motivation, concept mapping cut two ways: enthusiasm about the activity of concept mapping itself and motivation that came from working towards a goal. The multidisciplinary approach obviously motivated the experts: they expressed their surprise about the input of the others, and the disciplinary differences that became apparent. One of the expert groups decided to meet a third time because the gynaecologist did not agree with the internist’s viewpoint about how to categorize diagnoses. The pilot groups showed less motivation. Unlike the experts, the residents took the different viewpoints more for granted. They showed less motivation for the sessions. Although the target users (medical clerks) of the concept maps were described, it was especially the residents who remained uncertain about the level of knowledge of these target users.

“As clinicians you always concentrate on this part of the concept map (points to the patient-related concepts) but the most important piece of clinical reasoning is this (points to basic science concepts).” (E)

“Who is the target group?” (R)

Some groups expressed difficulties with the task to construct a concept map about all knowledge they considered absolutely relevant *for understanding* a clinical problem. The experts were inclined to create a decision tree, and thus seemed to be guided by the question: what knowledge does one use *for diagnosing* a clinical problem?

“I have trouble knowing where to start the thinking process.” (E)

#### Exchange of information

Explanations and motivations of contributions were clustered as ‘exchange of information’ (see Table 3). Participants explained how their disciplines coloured their views on the concepts.

“You think of the patient, as a first step, I think of the context.”(E)

Information was exchanged right from the start of the concept mapping process, when participants were collecting concepts. In the resident groups, these explanations already in this first stage often led to questions and hence interaction. This was the case in one expert group. There was only one resident group in which the emphasis in the communication was on exchange of information. In this group, one of the participants joined later.

#### Interaction

In the expert groups, interaction occurred in particular when joint decisions had to be taken, i.e., about links, the organization of the concepts and labelling the links. Although labelling the links was deemed unnecessary - most relations were causal or sequential - , it provoked discussions about what was cause and what consequence. The pilot group of two experts exhibited hardly any interaction. For residents, decision making was not a prerequisite to interact; most resident groups started interaction while collecting concepts, triggered by the contributions of the others. These contributions entailed concepts they did not know, leading to interaction, that is, asking for explanations or joint consultations of the Internet and remarks about their learning due to the input of others.

“What does MALT mean?” (R)

“I have added these concepts with another meaning in mind. Now, I discover that when you replace them, their meaning is changed.” (E) (indicating that by relating a concept to other concepts, meanings change somewhat)

Most resident groups spent much time organizing the concepts along the two structures offered: clinical concepts subsuming basic science concepts or the other way around, basic science concepts subsuming clinical concepts. Although some of them expressed having difficulties categorizing clinical concepts within a framework of basic science concepts, they all maintained a basic science categorization. This categorization provoked interaction: residents expressed doubts about which category to place some of the clinical concepts in, asked each other and consulted the internet.

“Is there a third group of pathophysiological explanations of proteinuria?” (R)

“I do not know whether this results in blood in faeces. You know that”. (R)

The instruction to analyse, summarize and explain the patient cases raised questions and therefore led to interaction. If a case did not belong to the domain of a particular participant, he/she tended to participate less in the discussion.

#### Decision-making process

The pilot groups of five participants had difficulties to reconcile five disciplinary viewpoints. Too many disciplinary viewpoints hindered the decision-making process about the organization of the concept map. This resulted in a bunch of concepts that to some extent were grouped, but not really organized or related.

“There are too many points of view. I don’t see how we can structure this”. (E)

“You seem to make a decision tree. But we are making a scientific ordering”. (E)

The residents started to order the concepts before they were instructed to do so. For them, an explicit instruction to consider different ways to order the concepts seemed to hinder rather than help decision making. Six of the seven expert groups did not start ordering until they received the instruction. They had frequently to be reminded to organize the concept map. All groups started with an ordering of the concepts that adhered closely to the phases of clinical reasoning (e.g., history, lab, diagnoses) and subsequently added an ordering as instructed: clinical concepts subsuming basic science concepts and basic science concepts subsuming clinical concepts. Basic science ordering sometimes evoked doubts about whether clinical or basic science concepts should be the organizational device, and slowed down decision making.

“In this schematization, you are trying to do two things at the same time: from basic science to differential diagnosis and from patient case to basic science knowledge. (R)

“The basic science categorization is good to know but you should not really apply it”. (E)

“But how do students learn? First anatomy, embryology. No, that does not work”. (E)

Decisions about linking concepts were based on considerations about complexity; too many links would make the concept map chaotic. In all groups, the instruction to analyse patient cases led to adaptations and helped to decide about the final version of the map. It was especially the residents who used the concept map for their own analysis and explanation of the case.

“Yes, I can reason along these lines” while pointing to a part of the concept map (R)

“Let’s stop expanding. Every concept covers more detailed concepts. This is a framework”. (E)

## Discussion

The multidisciplinary groups of medical teachers were able to articulate the integration of clinical and basic sciences in concept maps if they were guided by specific instructions. This ability was influenced by several factors. First, group size mattered: five disciplines in a group made decision making difficult. Optimal group size depends on the task [26], and for the task of constructing multidisciplinary concept maps three participants seemed optimal. Second, the learning process of these groups of three were found to be influenced by expertise level. Residents not only *articulated* integration of clinical and basic sciences to a greater extent (Vink SC, Van Tartwijk J, Bolk JH, Verloop N, Gosselink MJ: Consistent variations between concept maps constructed by expert groups and residents, submitted), they also *improved* their articulated integration to a greater extent, as the differences between draft and final versions show. In the cooperation between residents interaction was vital, whereas experts relied more on an exchange-of-information pattern. Taking the viewpoint of the theory of cooperative learning, which underscores interaction as a factor that affects learning, we assume that this interaction is a facilitating factor for the articulation of integration in the concept maps [22] and accounts for the higher degree of articulation in the residents’ concept maps. Moreover, decision making generated interaction in the expert groups. Whereas joint decisions and interaction are reported as two different factors that account for learning in cooperative learning settings in other studies [21,22], our results suggest that decision making and interaction are related: decision making turned out to be a means to induce interaction in the expert groups. In groups with too many participants decision making was rather difficult, probably due to too many gaps between disciplines that had to be bridged.

The extent of articulation of integration in concept maps could not explained by motivation, nor by the value participants attached to the procedure and the instructions. Our data suggest that it is the interaction provoked by the instructions focussing on integration that accounts for the articulation of integration. Both expert and resident groups expressed problems reconciling the two ordering devices in their concept maps. However, the experts remained focused on the clinical side of their explanation, described as chains of practice [1], whereas the residents used underlying basic science mechanisms for the organization of their maps more frequently, even though they experienced the instruction to order clinical concepts along the lines of basic science concepts as less helpful. We assume that it was not only the higher number of basic science concepts in the maps that accounted for residents’ prevalence for ordering along underlying basic science mechanisms, but that also the patho-physiological explanations residents gave each other might have caused them to focus on anatomical and pathophysiological explanations. This articulation of integration was further improved in the second session, an improvement which we could view as learning to articulate integration, similar to the learning processes in other concept mapping studies [14,25].

A combination of the cognitive, constructive and cooperative learning perspectives deepened our understanding of the use of concept mapping instructions on different expertise levels. The concept maps in our study not only disclosed characteristics of the shared knowledge of groups of clinicians and basic scientists on different expertise levels (which could be explained by cognitive psychological insights [4,30]) but also suggested that the articulation of integration can be improved, with the draft [version] functioning as a stepping stone for further articulation rather than a static reflection of cognitive structures. Hence, the ability to articulate integration might be considered a dynamic skill, subject to being influenced, as suggested elsewhere [13], and might thus be learned. The instructions appeared to affect the concept mapping process and subsequently the resulting concept maps, so that these maps, not surprisingly, differ somewhat from concept maps in other studies [6,14]. A striking difference is that most groups decided not to label the links, because overall these links indicated causal relations, which may be a consequence of the focus to linking in particular clinical and underlying basic science mechanisms. The cooperative learning view helped to detect decision making and interaction as facilitating factors for learning to articulate integration in expert and resident groups, and added another viewpoint to explain the differences between residents’ and experts’ maps. Cooperative learning is usually an approach for peer learning [21]. Both expert and resident groups were supposed to be peers: experts among experts and residents among residents. However, the definition of ‘peer’ might need to be differentiated. Experts might regard each other less as peers than residents do. Because of their specialized knowledge, the knowledge gap between experts might be larger than between residents. If this is the case, interaction might be a confounding variable for expertise level.

Our study has an explorative character and should therefore be continued in new experiments, for further refinement of the instructions and procedure. First, our results allowed us to state only the relatedness of interaction and articulation of integration. Follow-up research should examine whether there is a causal relationship. This might be investigated by triggering interaction in the expert groups by means of advancing decision making in the concept mapping session, and measuring the integration in the resulting concept maps. A next step is to quantify interaction and decision making, and correlate this to the integration articulated in the concept maps. Second, we conducted this study in one medical centre. Its specific organizational culture might have coloured the interaction between the experts and the way they have cooperated. This context variable should be taken into account in a follow-up study. Third, we endeavoured to detect patterns in the data from the video tapes and field notes, and decided to make a qualitative analysis with the risk of bias in the interpretation [28,29,31]. Such an explorative approach should be followed by research intended to quantify this qualitative information [32].

## Conclusions

For medical teachers learning to design educational programmes which reflect vertical integration, the question is how they can be instructed to articulate integration of clinical and basic sciences. Constructing concept maps in multidisciplinary groups of three has been found helpful. Participants’ motivation for concept mapping or their views on the instructions do not account for their ability to articulate integration. Factors that do affect medical teachers’ learning are process factors such as interaction and the need to make decisions. Influencing these factors by means of instructions could contribute to teachers’ ability to articulate relevant knowledge. When developing vertically integrated programmes, we should be aware of the gaps between disciplinary points of view on clinical problems. With more than three disciplines involved, we run the risk that integration remains confined to discussing separate viewpoints instead of relating them, a phenomenon that is also reported in PBL classes [33]. Finally, our results support the idea of involving residents in developing integrated curricula, because they are better able to articulate integration in concept maps and to question each other about the relevance of concepts and their relations than experts.
